# Near-Field Spectroscopy of Individual Asymmetric Split-Ring Terahertz Resonators

**DOI:** 10.1021/acsphotonics.3c00527

**Published:** 2023-08-03

**Authors:** Yuezhen Lu, Lucy L. Hale, Abdullah M. Zaman, Sadhvikas J. Addamane, Igal Brener, Oleg Mitrofanov, Riccardo Degl’Innocenti

**Affiliations:** † School of Engineering, New Engineering Building, Lancaster University, Gillow Ave, Bailrigg, Lancaster LA1 4YW, U.K.; ‡ Electronic and Electrical Engineering, University College London, London WC1E 7JE, U.K.; § Center for Integrated Nanotechnologies, Sandia National Laboratories, Albuquerque, New Mexico 87123, United States

**Keywords:** terahertz, metasurface, near-field spectroscopy, a-SNOM, plasmonics

## Abstract

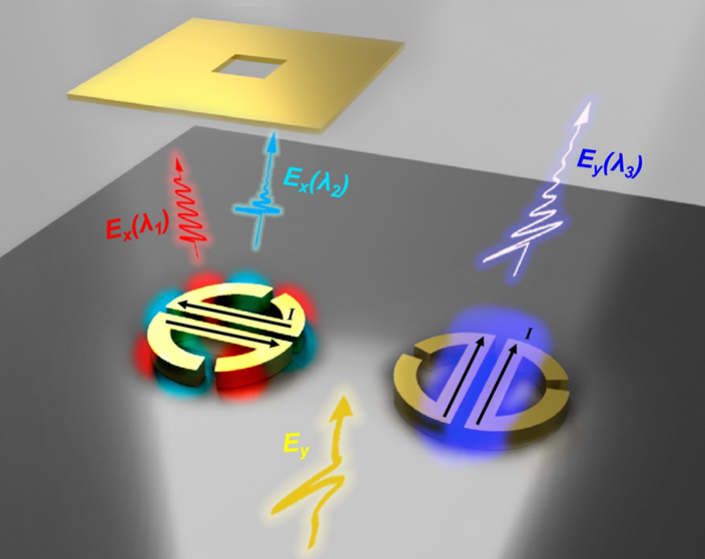

Metamaterial resonators have become an efficient and versatile platform in the terahertz frequency range, finding applications in integrated optical devices, such as active modulators and detectors, and in fundamental research, e.g., ultrastrong light–matter investigations. Despite their growing use, characterization of modes supported by these subwavelength elements has proven to be challenging and it still relies on indirect observation of the collective far-field transmission/reflection properties of resonator arrays. Here, we present a broadband time-domain spectroscopic investigation of individual metamaterial resonators via a THz aperture scanning near-field microscope (a-SNOM). The time-domain a-SNOM allows the mapping and quantitative analysis of strongly confined modes supported by the resonators. In particular, a cross-polarized configuration presented here allows an investigation of weakly radiative modes. These results hold great potential to advance future metamaterial-based optoelectronic platforms for fundamental research in THz photonics.

## Introduction

Research in the terahertz (0.1–10 THz corresponds to vacuum wavelengths between 30 μm and 3 mm) frequency range has led to impressive advancements in the key applications, including next-generation wireless communications,^1^ medical imaging,^2^ and sensing,^3^ as well as in the fundamental research in light–matter interaction,^4,5^ spintronics,^6^ and biological imaging.^7^ Many THz applications benefit from the implementation of resonators. For example, integration of resonators into THz modulators reduces the footprint and improves device efficiency.^8^ In compact THz sources, such as resonant tunneling diodes,^9,10^ quantum cascade lasers,^11−13^ and spintronic emitters,^14−16^ resonators can aid in the engineering of the photonic emission and improving the device performance in terms of lasing threshold, power consumption, and spectral versatility. High *Q*-factor resonators are also exploited for THz sensing as they yield higher sensitivity.^17−21^

The subwavelength nature of metamaterial (MM) resonators and their strong mode confinement can lead to enhanced light–matter interaction.^22−26^ At the same time, the strong mode confinement poses a challenge for direct experimental characterization of the modal properties. To overcome these hurdles, large arrays of such resonators are typically measured.^27^ However, these indirect measurements are acquired in the far-field and are affected by coupling between the array elements, as has already been discussed in previous studies.^28,29^ Furthermore, some modes, e.g., “dark” modes, prove to be elusive due to low visibility in the far field. Subwavelength resolution analysis of optical modes supported by THz MM resonators can be achieved by using scattering-type scanning near-field optical nanoscopy (conventionally named s-SNOM^30−38^) and by using aperture-type scanning near-field optical microscopy (a-SNOM).^39−43^ Near-field spectroscopy offers a unique tool for the investigation of quantum devices,^33^ in nanospectroscopy,^34^ and for the observation of plasmon polaritons^34^ also in graphene,^35^ topological insulators^37^ and phase change materials.^38^

Here, a THz broadband a-SNOM is used to study individual metallic asymmetric D-split-ring THz resonators (ADSRs). In this experimental configuration, a THz detector integrated with a subwavelength size aperture directly probes the evanescent electric field components of the modes supported by the metamaterial resonator. We combine the a-SNOM with a THz time-domain spectroscopy (TDS) analysis, which allows us to excite simultaneously all of the modes and to probe them selectively with the subwavelength spatial resolution of the near-field technique. We also apply a near-field probing configuration based on cross-polarized excitation and detection. It allows us to improve the visibility of the “dark” modes and to provide sharp near-field images of the deeply subwavelength resonators. The temporal evolution of the excited modes allows us to directly measure the *Q*-factors of single resonators with different geometrical sizes. The near-field experimental results are in excellent agreement with the predicted values, as well as with the complementary sets of measurements acquired in the far field with a commercial THz–TDS system. The ability to reveal the “dark” modes and associate spectral features with specific spatial distributions of strongly confined modes can aid in investigations of metamaterial resonators for THz applications.

## Results and Discussion

### Metamaterial Resonator Design

**Figure 1:**
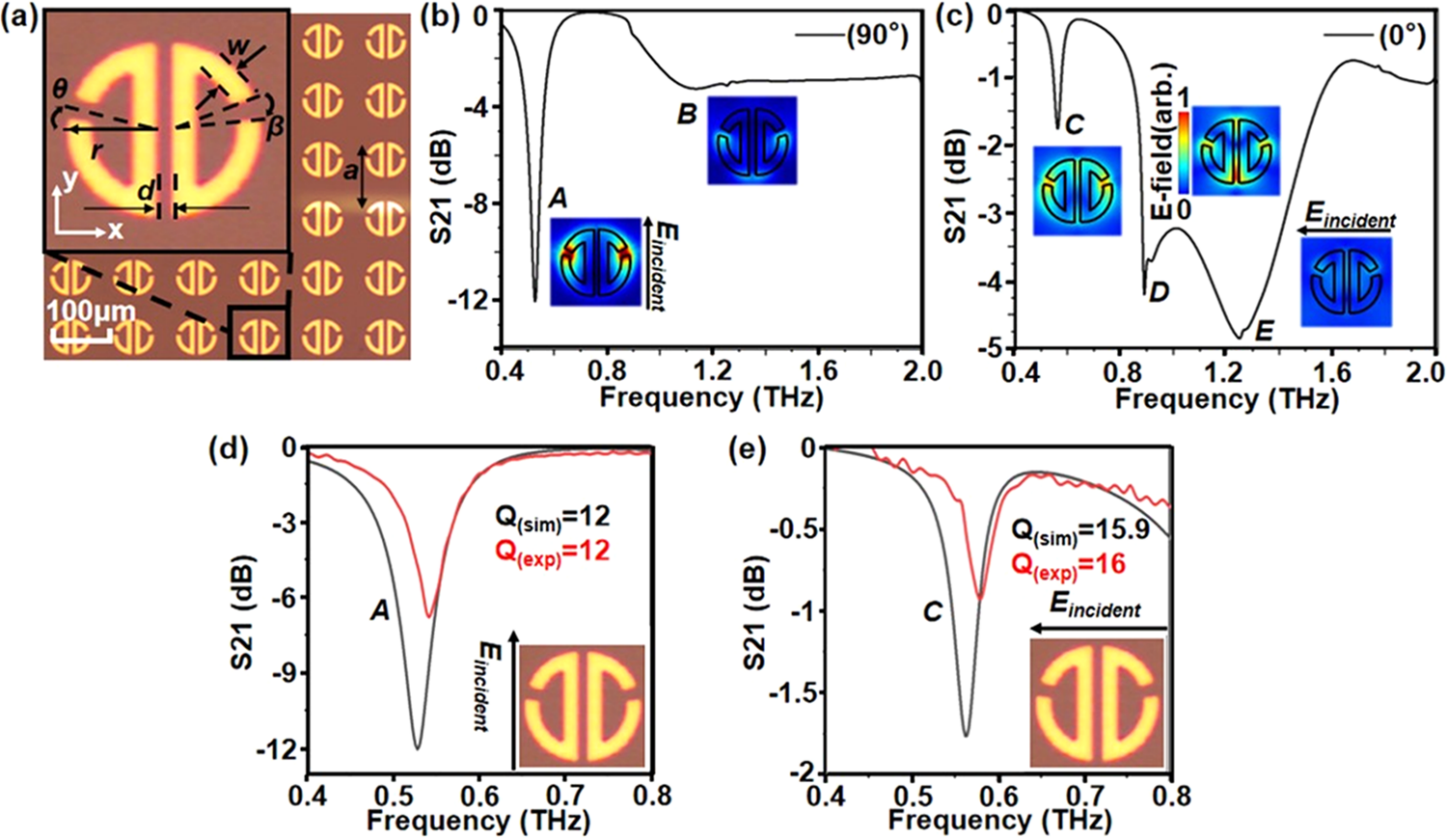
(a) Optical picture of the ADSR MM array resonator, inset: a unit cell illustrating resonator parameters. (b, c) Simulated far-field spectra for the 90 and 0° incoming E-field polarization, respectively. Insets: Simulated spatial profiles of the supported modes (normalized E-field at 2 μm above the ADSR surface). (d, e) Simulated and measured far-field spectra for modes **A** and **C**. Note the different vertical scales for each mode, which highlight the different mode visibility.

The resonator design is shown in Figure 1a. It is based on the ADSR first proposed in (44), composed of two equally separated ring halves with asymmetric gaps and two connect bars (further parameter details are reported in the Supporting Information). This specific design supports multiple modes when excited with light polarized in the *y*-axis (hereafter called 90°) or *x*-axis (hereafter called 0°), including dark modes arising from the asymmetric excitation of the currents in the linear segments of the upper and lower resonator halves. Such complex resonator designs based on the interplay between dark and bright modes have been realized to control the *Q*-factor values and therefore can be used to develop different functionalities, such as electromagnetic-induced transparency analogue^45,46^ and polarization modulators.^47^ However, the combination of dark and bright modes makes the ADSR challenging for far-field spectroscopy, and therefore, ADSR is an ideal candidate for near-field spectroscopic studies.

Figure 1b,c shows the broadband transmission spectra and normalized electric field profiles at resonances of an ADSR for two orthogonal incident polarization excitations, as simulated by using finite-element method commercial software COMSOL Multiphysics. Under THz illumination with the incident field polarized at 90°, a strong (“bright”) mode **A** originating from the circulating parallel currents is visible at ∼0.54 THz (S_21_ = −12 dB), while a weaker dipole mode **B** can be also observed at ∼1.15 THz (Figure 1b).

In contrast, under the 0° polarized incident E-field, the transmission spectrum shows three features corresponding to three resonant modes. The mode with the highest contrast (S_21_ = −5 dB) in Figure 1c is the dipole mode (**E**). The lowest frequency mode, mode **C**, exhibits much lower visibility (S_21_ = −1.7 dB). This mode originates from the antiparallel currents and can be considered as a “dark” mode. Mode **D** at around 0.9 THz is the mixed mode, showing characteristics from both modes **C** and **E**, with antiparallel currents in the central and dipole-like fields on the curved arms of the resonator.

### Far-Field Measurements

Preliminary measurements were performed in the far field using a commercial Menlo-K15 THz-TDS in transmission configuration to verify that the fabricated resonator arrays support these modes. The far-field measurements provide an experimentally solid mode mapping, which complements the numerical simulations and is needed in order to understand the near-field results. Further fabrication details are reported in the Supplementary Information in Table S1. Figure 1d,e presents the measured (red traces) and simulated (black traces) transmission curves of the resonances around 0.54 THz and 0.58 THz in the 90 and 0° polarization, corresponding to modes **A** and **C**, respectively. There is a very good agreement between the far-field measurements and the simulation curves. The visibility (here intended as the absolute value of the S_21_ parameter at resonance) of mode **A** (∼6 dB), simulated and experimentally measured, is significantly larger compared to the dark mode **C** (∼2 dB). The *Q*-factor of mode **C** is larger (*Q* ∼ 16) compared to the *Q*-factor of mode **A** (∼12). The experimental *Q*-factor was calculated by dividing the resonance central frequency by the FWHM, while the simulated *Q*-factor was directly provided by COMSOL Multiphysics.

### Near-Field Measurements

Following the far-field measurements, a second sample was fabricated on an identical substrate but with only 9 individual resonators (see Supporting Figure S1), spaced 600 μm apart and varied in size. Minor modification of the lithographic parameters was performed in order to match the resonators’ designs to the peak sensitivity of the near-field setup. The lithographic tuning allows us to modify the resonance frequency and the *Q*-factor of the resonators, without the need of fabricating several arrays for each design. These resonators show different far-field transmission spectra with respect to Figure 1 yet preserving the mode E-field characteristic distribution. The resonators were sufficiently spaced to measure the near-field response of a single resonator without interference from neighboring resonators. In the near-field setup, THz pulses were generated by an InAs source, which illuminates the sample from the substrate side at normal incidence. The near-field aperture probe was positioned ∼5 μm away from the front sample surface and kept fixed in all of the measurements reported (further measurements are reported in the SI). THz field detection is performed in a standard time-domain spectroscopy system using femtosecond pulsed NIR light from a Ti:Sapphire laser.^29^ The aperture probe consists of a gold planar surface with a 10 × 10 μm^2^ aperture, directly integrated on top of a photoconductive antenna detector (PCA). Therefore, fields which couple through the aperture are directly detected by the PCA behind the aperture plane. The field components that the aperture probe is sensitive to depend on the orientation of the PCA antenna—in this experiment, the antenna was oriented along the *y*-direction (parallel to the central bars of the resonators in Figure 1a), and the probe was sensitive to both the electric field polarized in the *y*-direction (more specifically, its time derivative is d*E*_y_/d*t*)^39^ and the out-of-plane electric field (more specifically, its spatial derivative with respect to the *y*-direction, d*E_z_*/d*y*).^39^ In contrast to the far-field transmission spectra, where a drop in amplitude is observed at the resonance, the near-field probe detects the evanescent field at the surface, and therefore, a stronger amplitude is observed at the resonance.

### Copolarized Configuration

The first measurements were focused on the resonator response for 90° polarized excitation. This is the simplest experimental configuration as the incident THz pulse in this polarization can be directly detected by the aperture probe. Furthermore, informed by simulations, we expect a clear strong spectral feature of mode **A** around 0.8–1.0 THz, where the near-field system has peak sensitivity.

**Figure 2:**
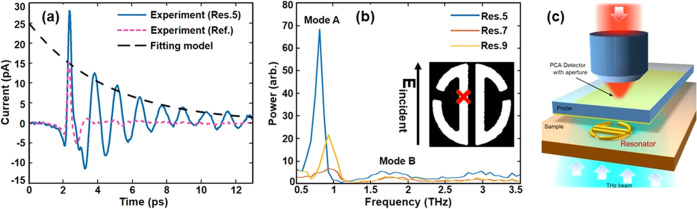
(a) Solid line: time-domain near-field waveform acquired at the center of resonator 5 with a-SNOM. Dashed purple line: reference time-domain waveform of the incident THz pulse, acquired in the substrate area (without the resonator). Dashed black line: best fitting curve for the evaluation of the *Q*-value. (b) Spectral amplitude of three isolated resonators (Res. 5, Res. 7, Res. 9), normalized to the spectrum measured in a region with no resonator present (pink dashed waveform in panel (a)). The inset shows a resonator sketch, indicating the position, where all of the waveforms were acquired. (c) Schematic of the near-field setup.

A typical time-domain pulse waveform measured near an example resonator (resonator 5) and a reference waveform measured on the substrate (no resonator) can be seen in Figure 2a. The time-delay stage step size is 5 μm corresponding to 0.033 ps. The spectral resolution is determined by the length of the time scan, which was cropped at about 12 ps (corresponding to a resolution of 30 GHz) at the time of the first reflections within the source and detector substrates in order to prevent Fabry–Perot fringes in the spectra. An optical image and lithographic parameters of this resonator are reported in the Supporting Information in Figure S1 and Table S2, respectively. Clear oscillations following the main pulse in the waveform can be attributed to the excitation of the “bright” mode (mode **A**). The far-field transmission measurement on resonator 5 for the 0° configuration (also supporting bright modes) is reported in the Supporting Information (Figure S3). Figure 2b shows the normalized spectra for three different resonators with varied ***d*** and β values (as defined in Figure 1a). As ***d*** or β increases, the resonance frequency and linewidth increase and the peak spectral amplitude decreases (as expected from simulations). Decreasing the ***w*** parameter also increases the *Q*-factor and the visibility. The resonance frequencies match those predicated by the simulations (see Supporting Figure S2). The time-domain waveforms detected by the a-SNOM can be used to directly evaluate the *Q*-factor of the resonance by using a fitting analytical model as shown in the dashed black line in Figure 2a (further details about the fitting function are reported in the Supporting Information).

**Figure 3:**
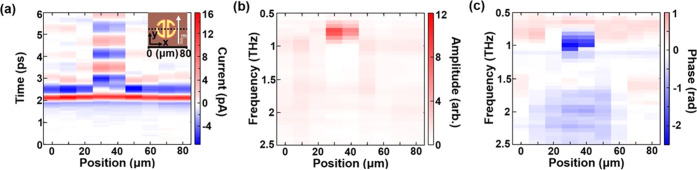
(a) Space–time scan across a single resonator (res. 5), with resonant oscillations observed at the resonator center. (b) Spectral amplitude at respective scan positions in panel (a). (c) Spectral phase at different probe positions, as illustrated in the inset, where the dashed line shows the probe position along the *X*-direction.

In this set of experiments, the detected signal was found to be highly sensitive to the probe position relative to the resonator. Figure 3a shows a space–time scan across a single resonator through the split-ring gaps (as shown in Figure 3c inset). Even at a short distance from the central bars of the resonator, the waveform shows a nearly single-cycle pulse, and no resonance excitation is detected. However, near the central bars, the THz waveform is altered drastically, and the resonant oscillations of the highest amplitude are recorded. Spatial maps were acquired at a fixed time delay for resonator 5, 7, and 9 (see the Supporting Information, Figure S6). All resonators show the highest signal near the central bars. In Figure 3b,c, we plot the power and phase spectra as functions of the near-field probe position. A clear spectral power peak and an abrupt change in phase are visible at around 0.8 THz for positions *x* = 30–40 μm (Figure 3b,c). The dependence of these spectral signatures on the probe position clearly demonstrates the advantage of the near-field technique: the near-field probe can isolate the THz signal in a 10 × 10 μm^2^ area, where the resonant field is the highest, drastically improving the mode visibility compared to far-field measurements.

### Cross-Polarized Configuration

**Figure 4:**
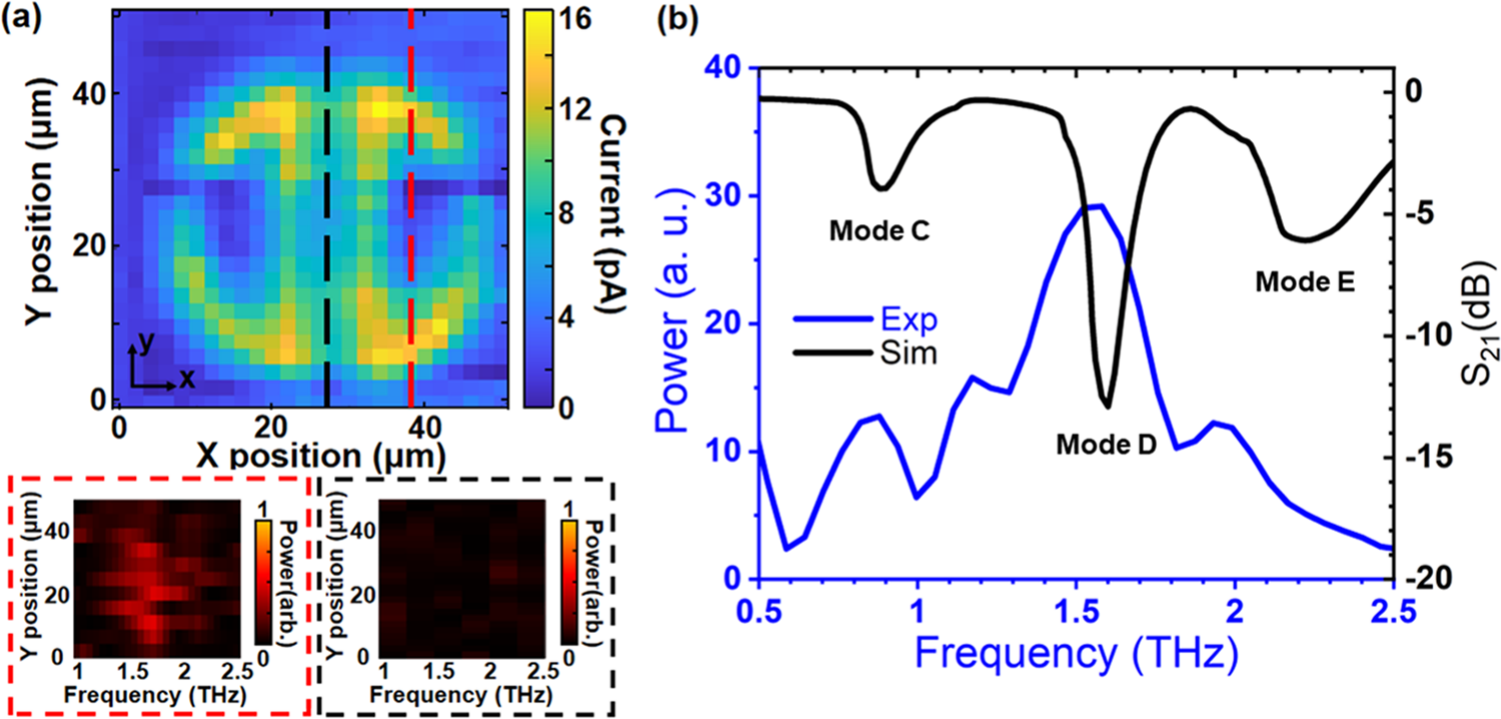
(a) Spatial map taken across resonator 5 at a time corresponding to a peak in resonant time-domain waveform. Red and black dash-framed insets: spectra of mode **D** in the frequency domain corresponding to the *Y*-axis scan at the yellow and black dashed lines in the spatial map, respectively. (b) Comparison between the averaged signal acquired through the whole resonator in the near-field experiment and the simulation results of the S_21_ parameter in the far field.

We now consider a near-field imaging and spectroscopic configuration, which inherently filters out the incident field polarization and detects only the orthogonal field components. Such a configuration can maximize the signal contrast due to the fields induced by the metallic structure of the resonator. In this configuration, the orientation of the probe and the resonator was kept fixed (thus, still measuring the *E*_y_ and *E*_z_ electric field components), while the incident field polarization was rotated to 0° (following the coordinate system shown in Figure 1). We selected resonator 5 from the previous section as this resonator gave the highest signal strength in the copolarized configuration (see Figure 2b). Figure 4a shows a near-field spatial map of the resonator acquired at a fixed time position corresponding to a peak in the resonant waveform. Remarkably, the 4 μm split-ring gap and central gap are clearly visible in the map, despite the 10 μm size of the aperture, which is larger than the resonator features. The near-field THz signal shows clear contrast between the metallic (gold) regions of the resonator and the substrate. We conclude that the probe is detecting the E-field components induced by the metallic lines at the Au/air boundary. The experimental map also shows similarities with the simulated field distributions for modes **C** and **D** shown in Figure 1c.

For spectroscopy of the resonator in this configuration, we recorded space–time maps taken along the *y*-direction (see Figure 4a) across the resonator and found that in contrast to the copolarized experiment, where the highest signal was found at the resonator center, the regions with the clearest resonance signature in the cross-polarized configuration were at the centers of the resonator rings. Therefore, time-domain waveforms were taken in the center of one of the rings (see red dashed line, Figure 4a). The average power spectrum is shown in Figure 4b (blue trace). The power is normalized to that of the 0° polarized incident field as it reflects the relative value of the detected field despite the fact that the excitation polarization is not directly detected in the cross-polarized configuration. The near-field spectrum, Figure 4b, shows distinct peaks at 0.8, 1.5, and 1.9 THz, which correspond in frequency to modes **C**, **D**, and **E** in the simulation presented in Figure 1. The very clear presence of modes **C** and **D** at 0.8 and 1.5 THz are particularly striking, given their antiparallel field profiles and therefore poor radiation into the far field. The spectrum demonstrates that the aperture probe can detect all three modes at the ring center.

We note that in contrast to the far-field spectra, the ratios of the mode amplitudes are different—with mode **D** (1.5 THz) being significantly stronger than those at 0.8 and 1.9 THz (presented in the Supporting Information in Figure S3). This apparent inconsistency comes from the fact that in the near field, the detected electric field represents contributions from all of the modes present at a specific probe location. Because of the difference in spatial distributions of these modes, the weighting of each mode’s contribution to the measured signal will depend on the probe position. To exemplify this, two insets in Figure 4a show the measured spectra in the frequency range of the dark mode **D** as a function of position across the resonator for two different line scans. When the probe is scanned across the ring, we see a clear resonant peak (red-framed inset). However, when the probe is scanned across the resonator center, this mode is no longer visible (black framed inset). This can be understood by considering the field distribution for the modes as shown in the simulations—the fields that we measured (*E*_y_ and *E*_z_ components) change most dramatically in the vicinity of the gaps in the resonator rings.

**Figure 5:**
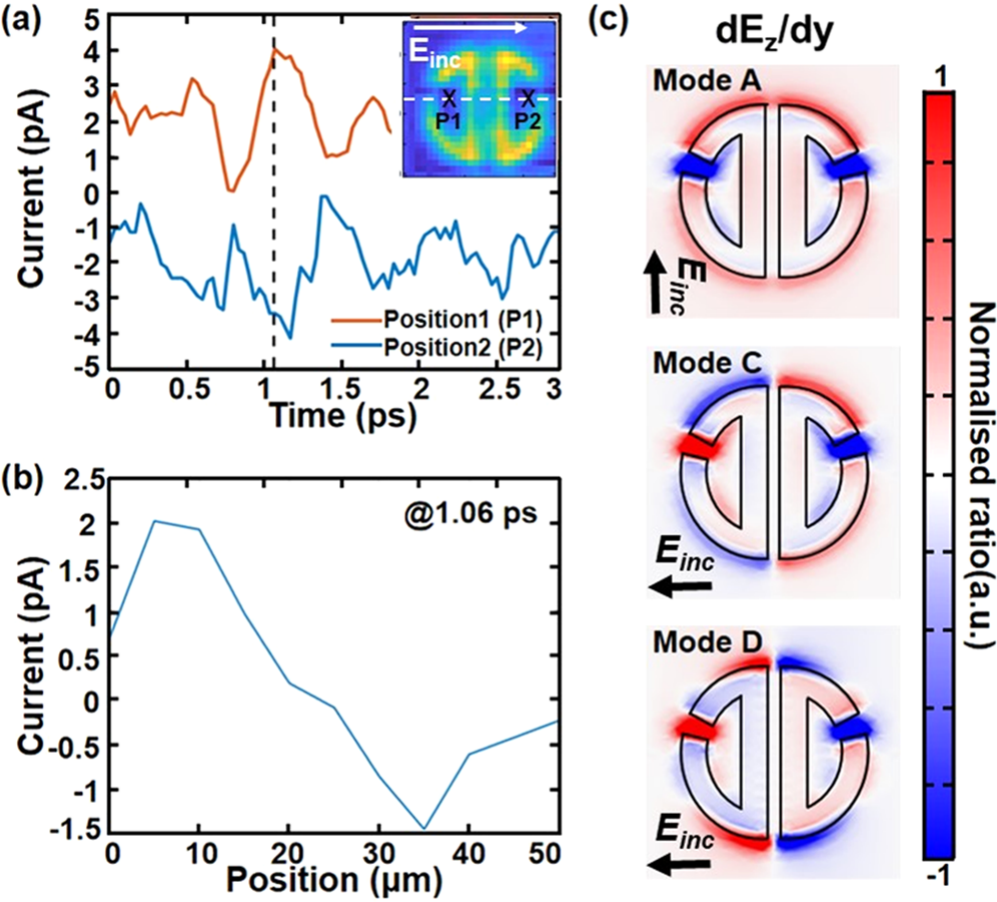
(a) Time-domain waveforms at different positions in resonator 5. Inset: Experimental map showing the scanning trace (white dashed line), position 1 and position 2 as described in the main text. (b) Amplitude changes of the waveforms at 1.06 ps according to the scanned positions in the resonator 5. (c) Simulations of d*E*_z_/d*y* in the ADSR for different modes. Clear signal flips can be found between the upper and the lower halves in modes **C** and mode **D**, while mode **A** does not show a phase change.

The phase of the near-field signal as a function of position can also help identifying “dark” from “bright” modes. The low signal amplitude in the cross configuration makes it difficult to reliably calculate the phase in a similar way to Figure 3c. Nevertheless, time waveforms measured at the two rings of the resonator show a distinct π phase change as shown in Figure 5a. Given the prominence of the **D** mode in the spectra, we deduce that the change of phase is largely a result of this mode. The phase change can be also seen in the scan along the *x*-position at *t* = 1.06 ps (Figure 5b). Our simulation of d*E*_z_/d*y* components for modes **C** and **D** confirms the antisymmetric field distribution for the two rings. This π phase change is a characteristic feature of “dark” modes in the ADSR. In contrast, mode **A** (“bright mode,” observed in the 90° polarization) shows a symmetric distribution (Figure 5c). Therefore, the a-SNOM technique is not only capable to clearly obtain the spectra of “dark” modes in individual resonators (Figure 4b) but can also pick out key characteristic features of these modes and differentiate them from standard dipole or radiative modes.

## Conclusions

This work demonstrates the unique potential and capabilities offered by THz a-SNOM for spectroscopic investigations of resonant modes supported by individual metallic ADSRs. We investigated single metamaterial resonators in the THz frequency range and obtained their *Q*-factors, avoiding inter-resonator coupling effects present in the far-field characterization of large arrays. We demonstrate that the a-SNOM technique is capable of capturing features of all of the supported resonant modes, including the highly confined, low-loss dark modes, in contrast to the standard far-field THz-TDS. Moreover, by implementing a cross-polarization measurement configuration, we found that the metallic features of the resonator can be mapped with unprecedented high-contrast and spatial resolution, better than expected from the size of the probe aperture. We also experimentally demonstrate the inherent difference between dark and bright modes in the spatial distribution of the corresponding field amplitude and phase. The near-field THz time-domain spectroscopy technique provides a unique tool for research ranging from development of novel THz optoelectronic platforms to investigations of exotic photonic phenomena, including bound state-in-continuum (BIC) resonances,^48^ which would benefit from advances in design and understanding of THz metamaterial resonators. Further insight in the working principle of these devices could be gained by using a monochromatic source, which would allow mapping of pure individual modes supported by the resonators without the inherent mode superposition that commonly occurs in excitation by broadband THz pulses considered here.

### Simulations

All of the simulations mentioned in this article were conducted by the finite-element method (FEM) via the RF module of Comsol Multiphysics commercial software. The simulation template is similar to the one used in (45, 46, 49). A single unit cell was simulated using lateral periodic boundary conditions to model the array, using the Drude model to describe the metallic parts and assuming a constant refractive index for Si of 3.418. An input E-field port and output port allowed the retrieval of the S_21_ parameter and the calculation of the *Q*-factor, as well as the E-field component mode distribution at the resonances. The actual simulation sizes can be referred to in the Supporting Tables S1 and S2.

### Fabrication

All of the metamaterial arrays shown in this article were fabricated on a 3 mm thick undoped Si substrate to avoid the Fabry–Perot effect in the far-field measurement and keep consistency in the near field. The total area of the sample used in the far-field measurement was 3 × 3 mm^2^, containing 30 × 30 unit cells. Both samples used in far-field and near-field structures were first started with standard laser writing techniques to pattern the shape of metamaterials, continued with the thermal evaporation of 10/150 nm Ti/Au on the Si surface and lift-off. The optical picture of the far-field sample is presented in Figure 1a, while a picture of the sample used for the near-field measurements is shown in the Supporting Information in Figure S1.

## Data Availability

The data underlying this study are openly available in Lancaster University repository PURE at DOI: 10.17635/lancaster/researchdata/612.

## Supporting Information Available

Additional experimental measurements and FEM simulations; optical images of the resonators; geometrical design parameters of the metamaterial resonators; theoretical frame and formulas used for the *Q*-factor calculation (PDF)
