# Supplementary material for: Near-Field Spectroscopy of Individual Asymmetric Split-Ring Terahertz Resonators
Source: ACS Photonics. 2023 Aug 3;10(8):2832–8. doi: 10.1021/acsphotonics.3c00527 (PMC10436345; doi:10.1021/acsphotonics.3c00527)
Supplement: Supplementary file 1 — ph3c00527_si_001.pdf [file ph3c00527_si_001.pdf]

Supplementary information

For the manuscript:

"Near-field spectroscopy of individual asymmetric split-ring Terahertz resonators"

***Yuezhen Lu, *Lucy L Hale, Abdullah M Zaman, Sadhvikas J Addamane, Igal Brener, Oleg Mitrofanov, Riccardo Degl'Innocenti**

This supplementary Information has 6 pages, 7 figures and 2 tables.

Resonator parameters

The main parameters for the resonators used in this work are reported in Table S1 and Table S2. Figure S1 shows an optical image of the 9 resonators sample used for the near-field measurements.

Table S1: Size of the 3*3 mm^2 far-field arrays corresponding to the measurement in Fig. 1 (d-e).

$\boldsymbol{r}$ (μm)	$\boldsymbol{d}$ (μm)	$\boldsymbol{\theta}$ (deg)	$\boldsymbol{w}$ (μm)	$\boldsymbol{\beta}$ (deg)	$\boldsymbol{a}$ (μm)
30.3	3.4	15	8.4	14	100

The parameters defining the resonators are showed in Figure 1 in the main text. The central gap $\boldsymbol{d}$, is the distance between the two halves, $\boldsymbol{r}$ is the radius of the outer ring, $\boldsymbol{w}$ is the width of the ring, $\boldsymbol{\theta}$ is the sweep angle of the center of the asymmetric gap from y -axis centre bar, $\boldsymbol{\beta}$ is the open angle of the gap and $\boldsymbol{a}$ is the periodicity of unit cells.

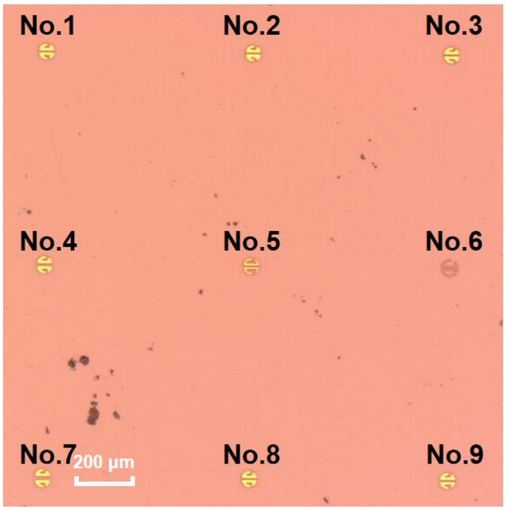

Figure S1: Optical image of the 9 different resonators prepared for the near-field measurement.

Table S2 Parameters of the 9 different resonators shown in Figure S1.

Resonator	r (μm)	d (μm)	θ (deg)	w (μm)	β (deg)
No.1		3.4	15	8.4	15
No.2		3.4	20	8.4	15
No.3		3.4	25	8.4	15
No.4		6	20	8.4	15
No.5	20.2	3.4	20	6	15
No.6		3.4	20	3	15
No.7		9	20	8.4	15
No.8		3.4	20	8.4	10
No.9		3.4	20	8.4	20

Finite element Simulations

Further normalized transmission (S21) simulations performed with Comsol Multiphysics for resonators 5, 7 and 9 are reported in Figure S2, together with the respective calculated Q factors for the mode **A**.

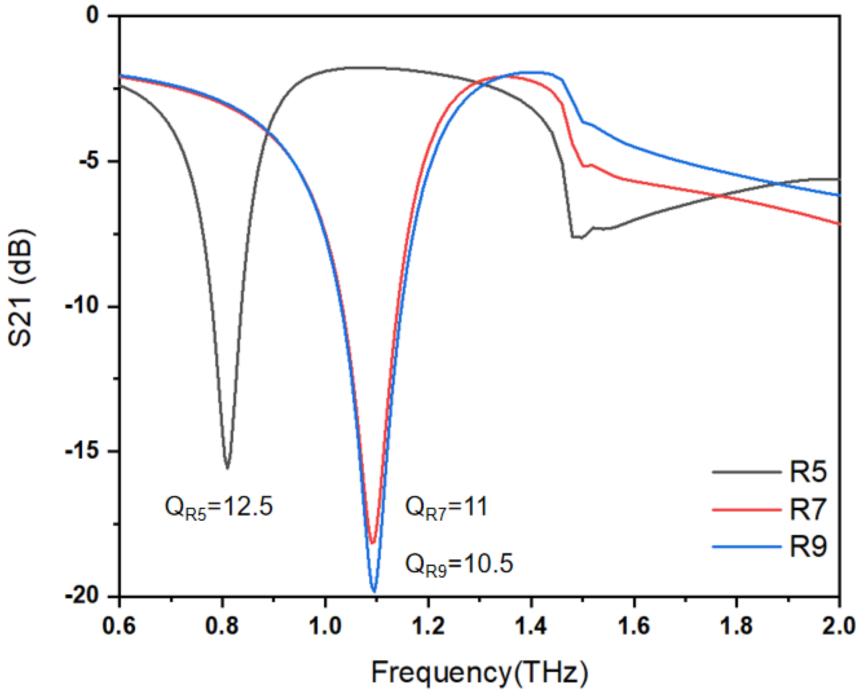

Figure S2: The S_{21} simulation of three different resonators (resonators 5,7 and 9) in the 90° polarized incident. The near-field experimental results show a minimal red shift in the central frequency compared to the simulations.

Far-Field measurements

Far-field transmission measurements for a 3x3 mm^2 array having as unit cell the same design of resonator 5 and a periodicity of 60 μm, for incident 0° incoming polarization are presented in Figure S3.

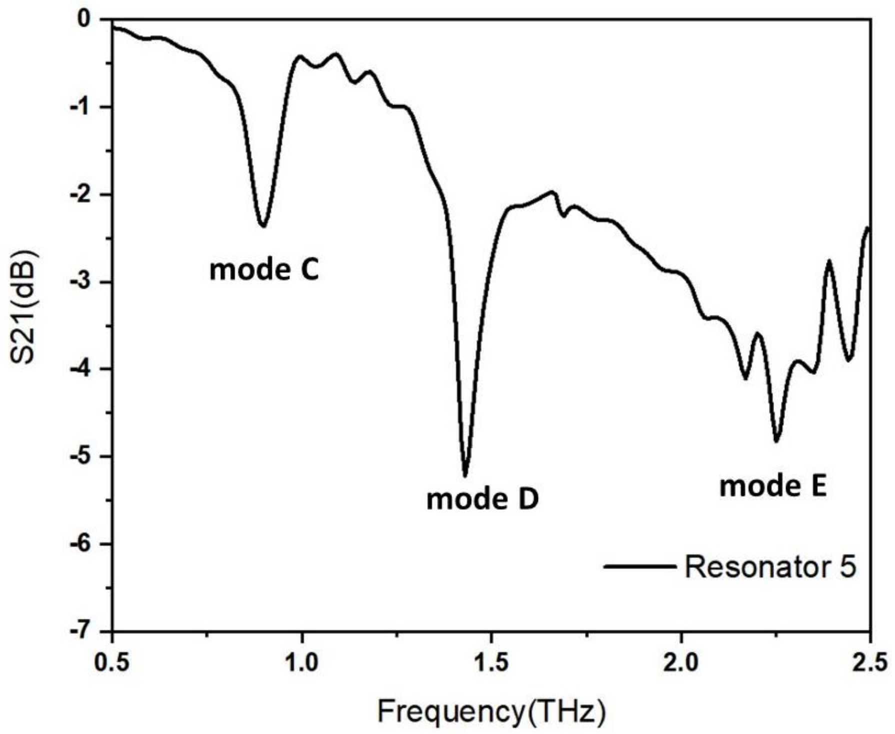

Figure S3: Far-field transmission measurement for resonator 5 array.

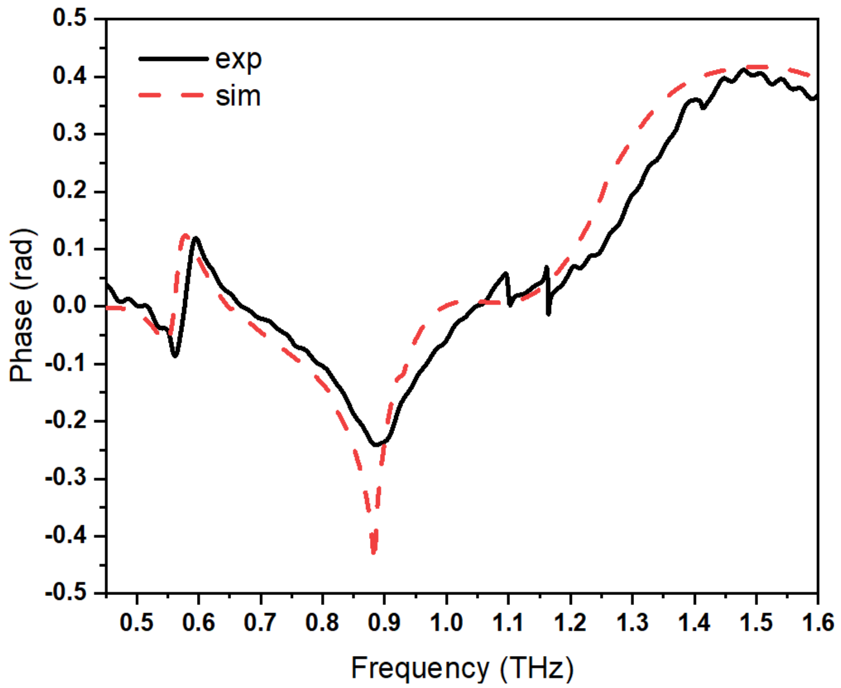

Figure S4: Simulated (red dashed line) and experimental (black solid line) far-field normalised phase.

The normalized far field phase change corresponding to the S_{21} simulation curve of Fig. 1 c) and the experimental far field trace in Fig 1 e) are reported in Figure S4. It is possible to clearly distinguish the dispersive features corresponding to the 0.55 THz and 0.9 THz resonance. The two features around 1.1 and 1.2 THz corresponding to water absorption lines, as the system was operating in open air.

Q factor measurements

There is a clear difference in both the spectral amplitude and Q-factor of the three resonators. The time-domain information from the individual resonances allows us to directly extract the resonance Q- factor using the single harmonic oscillation (SHO) model [1]. The classic SHO time domain waveform *y(t)* is:

$$y(t) = \alpha e^{-\gamma t} cos(\omega t - \varphi) \qquad (1)$$

Where α is amplitude, γ is decay rate, ω is the natural frequency of the oscillation. The *Q*-factor of the oscillation can be evaluated by:

$$Q = \frac{1}{2\xi} \qquad (2)$$

Where ξ is the damping ratio, it can be expressed as:

$$\xi = \frac{\gamma}{\omega} \qquad (3)$$

The experimental result and its exponential fitting of the resonator 5 are shown in Fig. 2(a), evaluating a *Q*-value of 11 ± 1 based on equations (1-3). The fitting procedure was performed without taking into consideration the first oscillation maximum in the E-field temporal waveform. Time-domain near-field waveforms acquired at the position specified in Figure 2 b) for resonators 5, 7 and 9 are reported in Figure S5 for completeness. The relative Fourier Transformed spectra are showed in the main text in Figure 2 b).

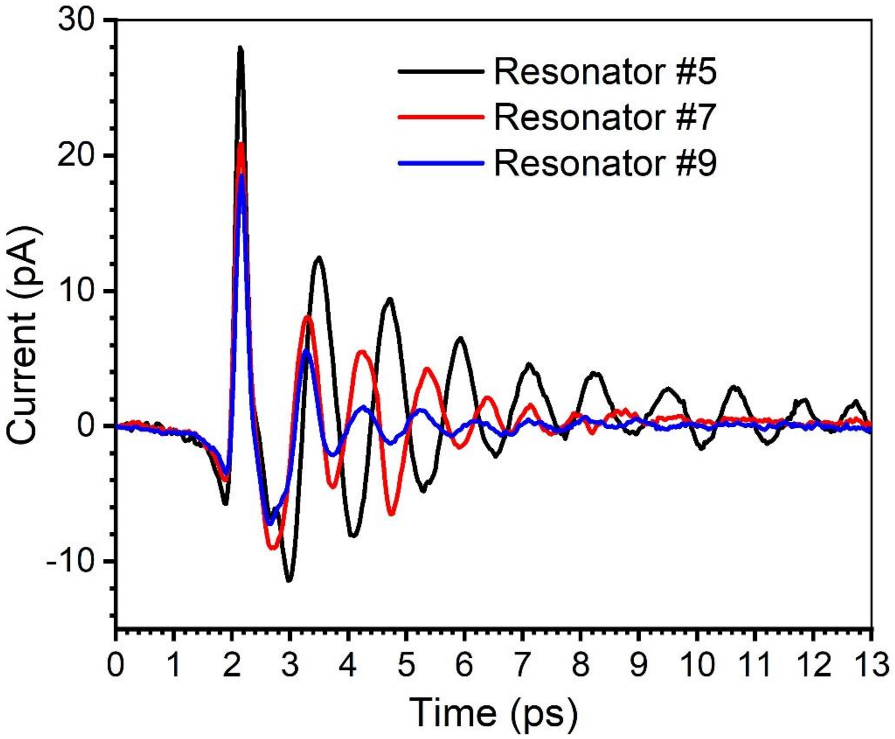

Figure S5: time-domain near-field waveform acquired at the center of resonator 5, 7 and 9.

Near-field Spatial mapping

Near-field spatial mapping for the 90° configuration are reported in Figure S6. The probe was scanned across the resonator area for resonators 5, 7 and 9, respectively reported in Fig. S6 a), b) and c). The normalized E-field for mode ***A*** is showed in the panel of Figure S6 d), together with the current density J. Finally, Figure S6 e) reports the time delay position (red dashed line) which was kept fixed during these measurements for resonator 5 (similar procedure was used for the other 2 resonators). It is possible to clearly observe the difference in the "d" parameter between resonators 7 and 9, as reported in Table 2.

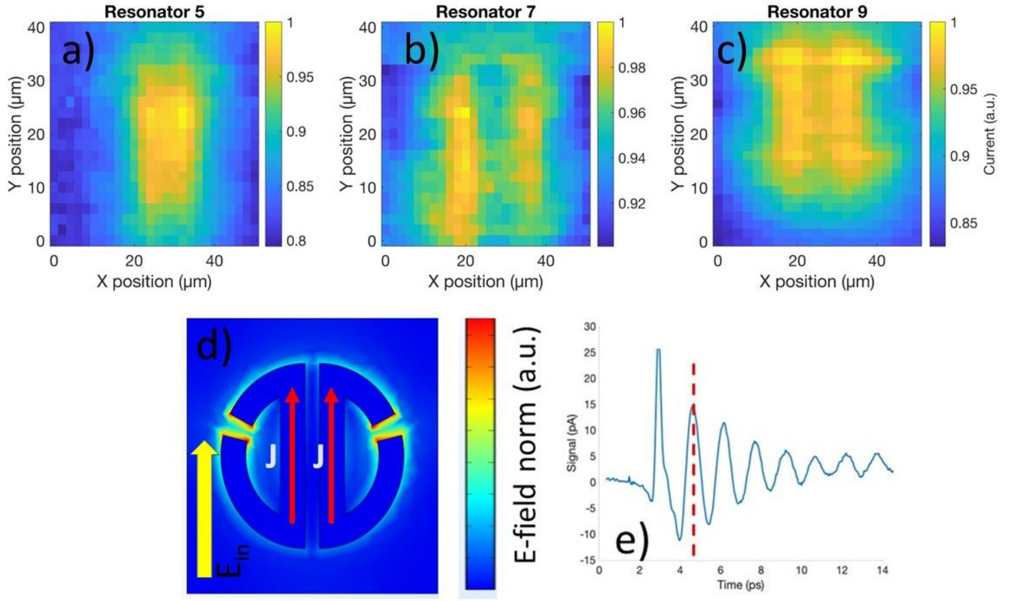

Figure S6: a)-c). Spatial mapping of resonator 5, 7 and 9. Mode A E-field norm distribution for resonator 5 and d) the relative fixed time delay used for the acquisition.

Sample-probe approach measurements

The sample-probe distance was estimated to be ~ 5 μm, as discussed in the main text and in [2,3]. An exemplar set of measurements acquired at different relative aperture/probe

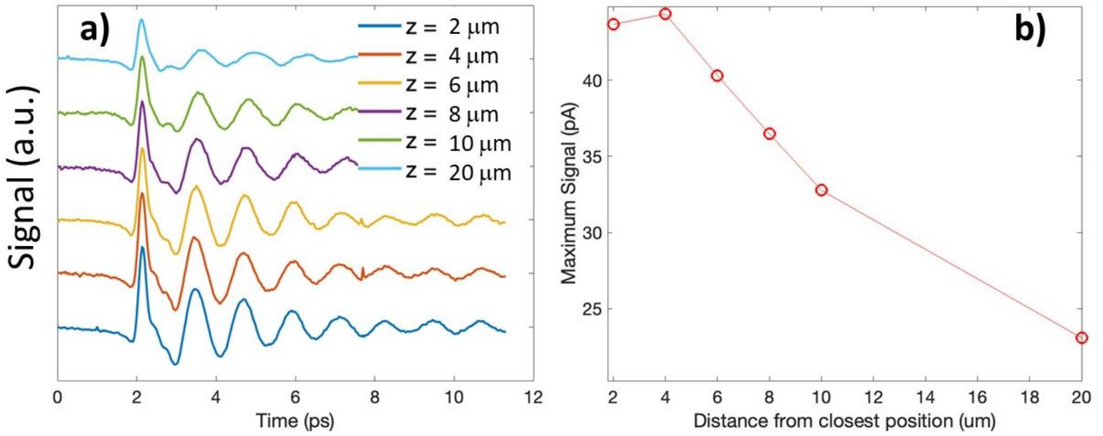

Figure S7: a) Time waveforms acquired on resonator 5 at different relative position z with respect to the minimal distance, allowing raster scanning. b) Maximum current signal recorded for different relative distances

distances z on resonator 5 is showed in Figure S7. It is possible to notice in Fig. S7 that the amplitude of the signal increases as the sample is brought closer to the probe. For all positions with z =2-10 μm, the waveform remains the same, and only the amplitude increases. Therefore, spectroscopic measurements can be performed at different distances within that range without affecting the results In this range, the resonant characteristics are not affected, but the maximum amplitude of the waveform reduces with increasing resonator-probe separation. The final spatial resolution is anyway limited by the probe dimension, which has size of 10x10 μm^2. We also note that there is no position feedback in our system and the position is controlled with ~1 μm precision.

References

[1] Hallauer,; W. Introduction to linear, time-invariant, dynamic systems for students of engineering 2016, Virginia Tech, A.T. Still University publisher, 2016.

[2] Hale, L. L.; Keller, J.; Siday, T.; Hermans, R. I.; Haase, J.; Reno, J. L.; Brener, I.; Scalari, G.; Faist, J.; Mitrofanov, O. Noninvasive Near-Field Spectroscopy of Single Subwavelength Complementary Resonators. Laser & Photonics Reviews 2020, **14**(4), 1900254.

[3] Khromova, I.; Kužel, P.; Brener, I.; Reno, J. L.; U-Chan Chung Seu, U.-C. C.; Elissalde, C.; Maglione, M.; Mounaix, P.; Mitrofanov, O. Splitting of magnetic dipole modes in anisotropic TiO_2 micro-spheres. Laser Photonics Rev. 2016, **10**, 681-687.
